# Keratinizing Pleomorphic Adenoma of the Buccal Mucosa: A Comprehensive Clinical and Histopathological Analysis of a Rare Case

**DOI:** 10.4314/ejhs.v34i3.10

**Published:** 2024-05

**Authors:** VK Varsha, HC Girish, Mamata Kamat, Vidya G Doddawad

**Affiliations:** 1 Department of Oral Pathology & Microbiology, Rajarajeshwari Dental College and Hospital, Bengaluru, Karnataka, India; 2 Department of Oral Pathology & Microbiology, Bharati Vidyapeeth (Deemed to be University) Pune, Dental College and Hospital, Sangli, Maharashtra. India; 3 Department of Oral Pathology and Microbiology JSS Dental College and Hospital A Constituent College of JSS Academy of Higher Education & Research, Mysuru, Karnataka

**Keywords:** Buccal Mucosa, Keratinizing pleomorphic adenoma, Minor salivary glands

## Abstract

Salivary gland tumors make up a relatively small proportion, ranging from 1% to 4%, of all neoplasms in the human body. Among these, pleomorphic adenoma stands out as a distinct benign tumor of the salivary glands, characterized by a combination of epithelial and mesenchymal elements.Only 0.3% to 1.5%, of biopsies in oral and Maxillofacial pathology laboratories are associated with tumors originating from minor salivary glands. Keratinizing pleomorphic adenoma, a rare variant accounting for 5-10% of all pleomorphic adenomas, differs from the typical form due to the presence of keratin within the tumor cells, serving as a distinguishing histological feature. The incidence of keratinizing pleomorphic adenoma is not well-established but is believed to be less than 1 case per 100,000 people per year. Here we present an atypical histopathological variation of pleomorphic adenoma, featuring extensive keratinization and manifesting in a less typical anatomical location in a 57-year-old male patient.

## Introduction

Salivary gland tumors make up a relatively small proportion, ranging from 1% to 4%, of all neoplasms in the human body. Among these, pleomorphic adenoma (PA) stands out as a distinct benign tumor of the salivary glands, characterized by a combination of epithelial and mesenchymal elements. The term “Pleomorphic adenoma” was coined by Willis, inspired by its unique light microscopic features, particularly architectural pleomorphism. Typically originating in major salivary glands, especially the parotid gland, it can occasionally arise in minor salivary glands (1). Only a small percentage, 0.3% to 1.5%, of biopsies in oral and Maxillofacial pathology laboratories are associated with tumors originating from minor salivary glands. Most tumors arising in intra-oral salivary glands tend to be malignant, benign tumors being rare (2).

These tumors are more prevalent in females, usually occurring between the 4th and 6th decades of life, and are seldom observed in children and adolescents. The morphological patterns of pleomorphic adenoma exhibit significant variability, including the presence of mucous cells, oncocytes, sebaceous cells, bone, adipose tissue, and crystalline materials (1). The occurrence of considerable squamous metaplasia, along with significant keratinization, is an infrequent observation in pleomorphic adenoma, especially within the minor salivary glands. In this case report, we present an atypical histopathological variation of pleomorphic adenoma, featuring extensive keratinization and manifesting in a less typical anatomical location in a male patient.

## Case Report

**Figure 1:**
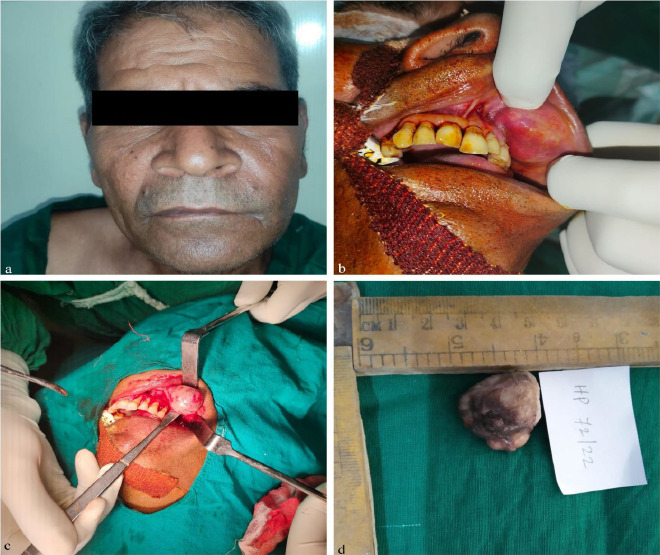
Extra oral Photograph of 57 years old Male patient (a); intra oral photograph showing lesion on left labial mucosa extending from 22 to 24 region (b); photograph showing intraoperative procedure (c); photograph showing gross specimen(d)

A 57-year-old male visited our dental clinic, reporting a painless swelling on the left buccal mucosa that he had observed for the past decade (Figure 1a). Over time, the swelling had gradually increased in size, occasionally causing discomfort during eating. The patient had an unremarkable medical history, with no reported history of radiation exposure or smoking. However, he disclosed a habit of betel quid (areca nut, slaked lime, and tobacco) chewing 3 to 4 times a day for 10 years.

Upon intraoral examination, a firm, non-tender, and mobile mass measuring approximately 3 cm x 3 cm in the left labial mucosa, extending from the 22 to 24 regions (Figure 1b) was noted. The overlying mucosa appeared intact, showing no signs of inflammation or infection. Facial nerve function was normal, and the salivary gland ductal opening appeared regular. No regional lymphadenopathy was observed.

**Surgical description**: Given the clinical presentation, a surgical excision was planned. The patient underwent an intraoral approach under general anesthesia. Careful dissection isolated the encapsulated and well-demarcated mass (Figure1c), which was completely excised with a margin of normal tissue to minimize recurrence risk. The partially encapsulated gross specimen was oval, whitish-grey in color, soft to firm in consistency, and measured about 2 cm × 2 cm × 2 cm (Figure 1d).

**Figure 2:**
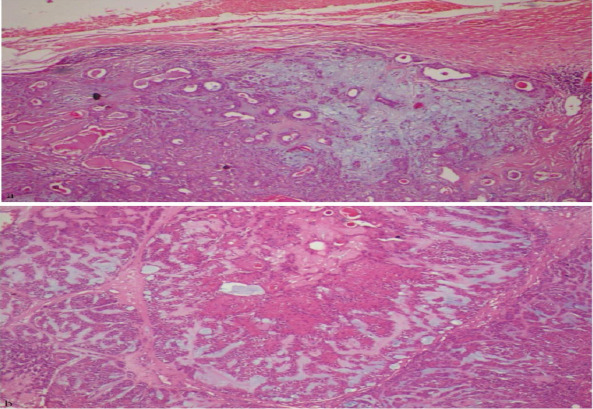
Photomicrograph showingpartial fibrous encapsulation surroundingmyxochondroid area with ductal component (a) (4x H& E);photomicrographshowing luminal and abluminal cells amidst myxoid stroma (b) (10 x H& E)

**Figure 3:**
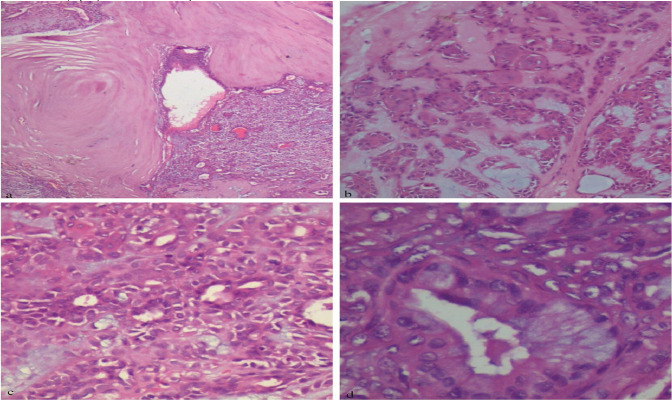
Photomicrograph showing extensive keratin pearl formation within lesional tissue (a) (20x H& E);photomicrograph showing squamous metaplasiaadmixed with tumour cells (b) (20 x H& E). Photomicrograph showing myoepithelial cells and ducts in myxoid stroma(c) (40x H& E) Photomicrograph showing mucous metaplasia of ductal epithelium (d) (40x H& E)

**Histopathological description**: Haematoxylin and eosin-stained sections of given specimen at a scanner view revealed a partially encapsulated circumscribed tumor (Figure 2a) with a cellular stroma consisting of ductal epithelial cells and chondromyxoid areas (Figure 2b). Numerous cystic spaces containing large keratin pearls were evident (Figure 3a). Under higher magnification, architectural patterns such as ducts composed of luminal and abluminal elements amidst myxoid stroma were observed (Figure 3b), along with focal areas of extensive keratin pearls (Figure 3c) and mucous metaplasia (Figure 3d).

## Discussion

PA is the most prevalent salivary gland tumor, comprising 60% of cases, with a predominant occurrence on the palate (65%) and lesser occurrences on the cheek (15%), tongue, floor of the mouth, and rare involvement of the lip (less than 1%). Histopathologically, PA displays diverse morphology arising from interactions between its cellular and stromal components, showcasing sebaceous, lipocytic, and oncocytic metaplasia in both stromal and epithelial element (3). The myoepithelial cell plays a crucial role in determining the general composition and appearance of a mixed tumor, and one of its distinctive characteristics is the potential for extensive squamous differentiation (1).

Keratinizing pleomorphic adenoma (KPA), constituting 5-10% of all PAs, distinguishes itself from the typical form through the presence of keratin within tumor cells, a distinctive histological feature.(4) The incidence of KPA is not well-established but is believed to be less than 1 case per 100,000 people per year.(5) To the best of our knowledge, the review of the English literature till 2023 revealed a total of 3 cases of KPA in Minor (Table 1). Squamous metaplasia and keratin pearl formation in PA result from acinar cell dedifferentiation, followed by hyperplasia of acinar, duct luminal, and myoepithelial cells. Focal squamous metaplasia in PA may be related to factors like ischemia, tissue repair post-infarction, and salivary gland necrosis (4). Keratin pearls in pleomorphic adenoma are commonly observed in intra-oral salivary glands rather than major glands, suggesting that the epithelium of minor salivary gland ducts, exposed to various irritants, may contribute to their development (5).

**Table 1:** Summary of previous reported cases of KPA in minor salivary glands

	Author	Age/sex	Site	Duration	No of cases	Recurrence
1	Tandon A et al 2018.(5)	28/M	Left palate	7-8 yrs	1	NED -6 months
2	Anjum R et al 2019.(4)	45/M	Right labial mucosa	3yrs	1	NED -6 months
3	Leena Sankari et al 2021.(5)	36/M	Left Buccal mucosa	1 yr	1	NA

In a specific case, betel quid chewing was identified as a potential source of irritation, although the exact underlying mechanism remains unclear. The unique combination of localization, gender, and microscopic features in this case distinguishes it from the typical presentation of PA. The presence of keratin pearls in PA can pose a diagnostic challenge, resembling malignancies such as epidermoid carcinoma, adenoid squamous cell carcinoma, and mucoepidermoid carcinoma. The significance of KPA lies in its potential misdiagnosis as a malignant tumor, leading to unnecessarily aggressive treatment.^5^ Careful assessment of the cytologic features like lack of dysplastic features, presence of duct-like areas, and mesenchymal components helps to differentiate it from similar lesions and arrive at the diagnosis.

In conclusion, salivary gland tumors constitute a small percentage of neoplasms, with PA being a prevalent benign tumor characterized by epithelial and mesenchymal elements. This case report highlights an atypical presentation of PA in a male patient with a history of betel quid chewing. The histopathological examination revealed a KPA, a rare variant accounting for 5-10% of all PAs, featuring extensive keratinization. The unique combination of clinical, demographic, and microscopic characteristics in this case distinguishes it from the typical presentation of PA. The presence of keratin pearls poses a diagnostic challenge, emphasizing the importance of careful assessment to avoid misdiagnosis and unnecessary aggressive treatment.
